# Mean Corpuscular Volume as a Predictive Factor of Response to Preoperative Chemoradiotherapy in Locally Advanced Rectal Cancer

**DOI:** 10.1155/2018/6976375

**Published:** 2018-03-18

**Authors:** Wen-Yi Zhang, Xing-Xing Chen, Wen-Hao Chen, Hui Zhang, Chang-Lin Zou

**Affiliations:** Department of Radiotherapy and Chemotherapy, The First Affiliated Hospital of Wenzhou Medical University, Wenzhou, China

## Abstract

**Background:**

The aim of this study was to identify if blood routine parameters and serum tumor marker are potential predictive factors for tumor response to preoperative chemoradiotherapy (CRT) in locally advanced rectal cancer.

**Materials and Methods:**

55 locally advanced rectal cancer patients were treated with preoperative CRT in this study. The total dose of preoperative radiotherapy was 45 Gy in 25 fractions of 1.8 in 5 weeks. All patients concurrently received 825 mg/m^2^ capecitabine orally twice daily on days 1 to 14 and 22 to 35. Total mesorectal excision (TME) was performed 6 weeks after the end of preoperative CRT. Blood routine examination and serum tumor marker were checked before preoperative CRT. Tumor response to preoperative CRT was evaluated with the semiquantitative tumor regression grading (TRG) system proposed by Dworak criteria according to histopathological examination of the surgical specimens. Univariable and multivariable logistic regression analyses were used to test the association between blood routine parameters and serum tumor marker and tumor response to preoperative CRT.

**Results:**

Univariate logistic regression analysis revealed that differentiation, lymphocyte, LMR, MCV, PLR, and CEA have been significantly associated with tumor response to preoperative CRT. Multivariate logistic regression analysis revealed that differentiation, MCV, and CEA were the predictors of tumor response to preoperative CRT. According to the ROC analysis, the AUC of differentiation, MCV, and CEA was 0.794, 0.802, and 0.723, respectively. Optimal cutoff points for MCV and CEA were 87.65 fl and 4.05 ng/ml, respectively.

**Conclusion:**

MCV is a potential predictive factor for tumor response to preoperative chemoradiation in locally advanced rectal cancer.

## 1. Introduction

Rectal cancer is one of the most common cancers in the world [1]. For patients with locally advanced rectal cancers (stage II to III), preoperative chemoradiotherapy (CRT) followed by radical surgery is currently used worldwide and considered the gold standard regimen [2]. Pathological complete response (pCR) and pathological partial response (pPR) rates in locally advanced rectal cancer patients with preoperative CRT followed by radical surgery are approximately 25% and 60%, respectively [3]. However, for patients with stable disease (SD) or progressive disease (PD), this treatment regimen may have disadvantages such as promoting disease progression or delaying surgery and meanwhile affected resectability, local control rates, disease-free survival, and overall survival [4].

This study attempts to assess the relationship between pathological reaction and clinical factors and blood routine parameters in patients with locally advanced rectal cancer who received preoperative CRT followed by radical surgery.

## 2. Materials and Methods

### 2.1. Patients

This retrospective study was conducted using the database from the patients with stage II to III rectal cancer who received preoperative CRT followed by radical surgery from 2014 through 2016. This study was approved by the Ethics Committee of the First Affiliated Hospital of Wenzhou Medical University. The eligibility criteria were as follows: preoperative CRT, radical surgery, and magnetic resonance imaging (MRI) before treatment. The MRI and endorectal ultrasound (ERUS) were performed before preoperative CRT for staging of the patient according to the TNM system. Postoperative restaging was based on pathological report.

### 2.2. Preoperative Chemoradiotherapy

All patients received preoperative radiotherapy which consisted on 45 Gy in 25 fractions of 1.8. Three dimensionally planned conformal radiotherapy (3D-CRT) was planned for each patient. The radiation fields included one posterior field and two lateral fields. The superior border of those three fields was at the L5/S1 level. The inferior border of those three fields was 2-3 cm below the tumor or at the level of obturator foramen. The lateral borders of the posterior field have a 1.5 cm margin beyond the true pelvic sidewalls. The external iliac nodes were not included in the radiation fields. All patients received 825 mg/m^2^ capecitabine orally twice daily on days 1 to 14 and 22 to 35. Total mesorectal excision (TME) was performed 6 weeks after the end of preoperative radiochemotherapy.

### 2.3. Treatment Evaluation

Tumor response to preoperative CRT was evaluated with the semiquantitative tumor regression grading (TRG) system proposed by Dworak criteria [5] according to histopathological examination of the surgical specimens. TRG ranges from 0 to 4: TRG 0 as no regression, TRG 1 as dominant tumor mass and obvious fibrosis in less than 25% of the tumor mass, TRG 2 as dominant tumor mass with obvious fibrosis in 26%–50% of the tumor mass, TRG 3 as dominant fibrosis outgrowing the tumor mass, and TRG 4 as complete regression [5, 6]. The “response group” was defined as TRG 3 or TRG 4, and the “no response group” was defined as TRG 0, TRG 1, or TRG 2 [7].

### 2.4. Statistical Analysis

Blood routine examination, serum carcinoembryonic antigen (CEA), serum alpha fetoprotein (AFP), and serum carbohydrate antigen 199 (CA199) were checked before preoperative CRT. The potential predictive factors were as follows: age, sex, differentiation, white blood cell count (WBC), neutrophil granulocyte, monocyte, lymphocyte, eosinophilic granulocyte, basophilic granulocyte, neutrophil-to-lymphocyte ratio (NLR), lymphocyte-to-monocyte ratio (LMR), neutrophil-to-monocyte ratio (NMR), hemoglobin, hematocrit (HCT), mean corpuscular volume (MCV), red cell distribution width (RDW), platelet, platelet-to-lymphocyte ratio (PLR), thrombocytocrit, CEA, AFP, and CA199. Chi-square tests were used for analysing correlation between the predictive factors and tumor response. The univariate analysis and multivariate analysis were performed by the logistic regression analysis to determine the significant predictors of tumor response to preoperative CRT. The receiver operating characteristic (ROC) curve analysis was used to calculate the area under the curve (AUC) and check the value of the statistically significant variables (*p* < 0.05). All data were analysed using SPSS 18.

## 3. Results

10 (18.2%) patients were considered to have complete regression (TRG 4). TRG 3 was observed in 16 patients (29.1%). The “no response group” included 15 (27.2%) patients with TRG 2, 12 (21.8%) patients with TRG 1, and 2 (3.6%) patients with TRG 0. 5 patients (9.1%) and 4 patients (7.3%) were female in the “response group” and the “no response group,” respectively. And the median age in the “response group” and the “no response group” was 60 years and 57 years, respectively. All patients' tumors was adenocarcinoma. Patient characteristics and blood routine parameters were shown in Table 1.

**Figure 1:**
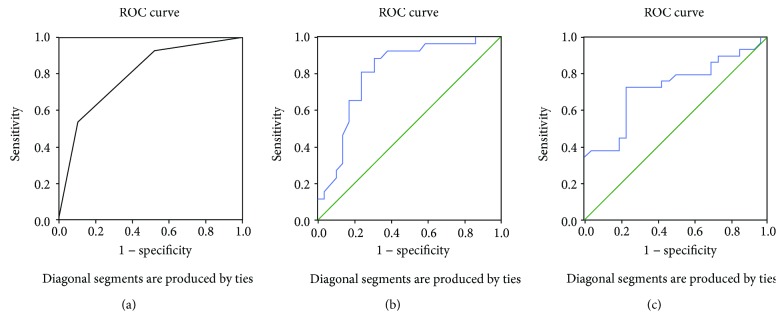
Receiver operating characteristic (ROC) curve plotted to check the value of a statistically significant variable in the logistic regression model for differentiation (a), MCV (b), and CEA (c).

Univariate logistic regression analysis revealed that differentiation, lymphocyte, LMR, MCV, PLR, and CEA have been significantly associated with tumor response to preoperative CRT. Multivariate logistic regression analysis revealed that differentiation (OR 0.056 (95% CI 0.004–0.889), *p* = 0.041), MCV (OR 0.615 (95% CI 0.401–0.942), *p* = 0.025), and CEA (OR 1.639 (95% CI 1.126–2.386), *p* = 0.010) were the predictors of tumor response to preoperative CRT. Tables 2 and 3 show the logistic regression model for prediction of tumor response to preoperative CRT. According to the ROC analysis, the AUC of differentiation, MCV, and CEA was 0.794, 0.802, and 0.723, respectively. Optimal cutoff points for MCV and CEA were 87.65 fl and 4.05 ng/ml, respectively. The ROC curve is shown in Figure 1.

According to the optimal cutoff point of MCV, the patients were classified into two groups, the high MCV and low MCV groups. It is found that patients in the “response group” were the majority of the high MCV group (88.46% versus 11.54%) and patients in the “no response group” were the majority of the low MCV group (65.52% versus 34.48%).

According to the optimal cutoff point of CEA, the patients were classified into two groups, the high CEA and low CEA groups. It is found that patients in the “response group” were the majority of the low CEA group (76.92% versus 23.08%) and patients in the “no response group” were the majority of the high CEA group (72.41% versus 27.59%).

## 4. Discussion

In this study, there was a significant association between elevated levels of MCV and good tumor response to preoperative chemoradiation in advanced rectal cancer. There was a significant association between decreased levels of CEA and good tumor response to preoperative chemoradiation. Additionally, remarkable significance was reached for differentiation. The tumor with low differentiation had good response to preoperative chemoradiation. The percentage of patients in TRG 0, TRG 1, TRG 2, TRG 3, and TRG 4 in our study was approximately close to other studies [6, 7].

Dellapasqua and colleagues [8] reported that elevated MCV was related to decreased risk of disease progression in patients treated with chemotherapy for metastatic breast cancer. Cokmert and colleagues [9] reported that increased MCV may be used as a predictor of improved progression-free survival (PFS) and overall survival (OS) in patients with metastatic colorectal cancer who were treated with capecitabine. Jung and colleagues [10] reported that there was a significant association between higher MCV and longer PFS and OS in patients with advanced gastric cancer who were treated with capecitabine. The studies mentioned above have focused on the relationship between MCV and PFS and OS. And this study found that higher MCV level was associated with good tumor response to preoperative CRT. There was a significant correlation between MCV and several oxygen parameters [11]. Higher MCV are associated with an elevated oxygen pressure [11] and an increased oxygen affinity in red blood cells [12]. And then higher MCV leads to enhanced oxygen saturation in red blood cells. Therefore, higher MCV may facilitate oxygen delivery [13]. The amount of oxygen released from red blood cells into the tumor tissue increased. The increased total oxygen content inside the tumor tissue resulted in the reduced ratio of hypoxic tumor cell playing an important role in chemoradioresistance [14]. Finally, there is an increasing chemoradiosensitivity in tumor tissue.

Some studies reported that CEA level is a predictor of tumor response to preoperative CRT in rectal cancer. Das et al. [15] reported that CEA level, circumferential extent of tumor, and distance from the anal verge may be used to predict the pathologic response to preoperative chemoradiation for patients with rectal cancer. Park et al. [16] reported that elevated serum CEA levels in rectal adenocarcinoma patients are associated with poor response to CRT. Park and colleagues [17] have evaluated the relationship between serum CEA and tumor response in rectal cancer patients treated with preoperative CRT. They found [17] that the good response was significantly associated with the lower level of pre-CRT CEA. Restivo and colleagues [18] noticed that there was a significant correlation between serum CEA lower than 5 ng/dl and complete pathological response after preoperative treatment in patients with rectal cancer. Similarly, this study found that serum CEA level in patients with good tumor response to preoperative CRT was significantly lower than that in patients with no response.

Benej and colleagues [19] reported that left upper lobe non-small cell lung cancer patients with WBC lower than 10 × 10^9^/l on the third day after the operation had significantly higher overall survival than peer with WBC count higher than 10 × 10^9^/l. Peng and colleagues [20] reported that there was a significant association between neutrophil, monocyte, lymphocyte, red blood cell count, NLR, LMR, and risk of colorectal cancer mortality. Taussky and colleagues [21] reported that a posttreatment high WBC and lymphocyte count increases the overall mortality of localized prostate cancer patients receiving radiotherapy. Li and colleagues [22] reported that preoperative high NLR was associated with poor recurrence-free survival in patients with epithelial ovarian cancer. Qin and colleagues [23] reported that patients with ovarian cancer had significantly absolute neutrophil count, NLR, and PLR than normal population. However, we did not identify that WBC, NLR, and LMR could predict tumor response to preoperative CRT.

Wei and colleagues [24] reported that higher RDW levels were significantly higher in the gastric cancer patients than in normal population. Ay and colleagues [25] reported that RDW levels in patients with colon cancer were significantly higher than its level in patients with colon polyp. Kust and colleagues [26] reported that RDW levels both pre- and postoperative had significant association with overall survival in patients with colorectal cancer. Li and colleagues [22] reported that preoperative high RDW was associated with poor recurrence-free survival in patients with epithelial ovarian cancer. Qin and colleagues [23] reported that patients with ovarian cancer had significantly higher level in the RDW than normal population. However, we did not identify that RDW could predict tumor response to preoperative CRT.

## 5. Conclusions

Our research identified MCV as a potential predictive factor that may allow personalization of preoperative CRT in locally advanced rectal cancer. However, more investigation and larger samples of patients in locally advanced rectal cancer are needed further to confirm the relevance of this result.

## Figures and Tables

**Table 1:** Patient characteristics.

Variable	Response (26)	No response (29)	*p*
Sex			
Female	5	4	0.721
Male	21	25	
Age (y)			
Median (range)	60 (38–88)	57 (31–87)	0.145
Differentiation			
Low	14	3	<0.001
Moderate	10	12	
High	2	14	
White blood cell count (×10^9^)			
Median (range)	6.20 (3.89–12.50)	6.46 (3.27–12.44)	0.416
Neutrophil granulocyte (×10^9^)			
Median (range)	3.62 (2.36–10.20)	4.20 (1.79–10.70)	0.434
Monocyte (×10^9^)			
Median (range)	0.50 (0.26–0.90)	0.59 (0.15–1.07)	0.142
Lymphocyte (×10^9^)			
Median (range)	1.91 (0.80–3.90)	1.40 (0.80–3.02)	0.017
Eosinophilic granulocyte (×10^9^)			
Median (range)	0.11 (0–0.50)	0.14 (0–0.44)	0.195
Basophilic granulocyte (×10^9^)			
Median (range)	0.015 (0–0.10)	0 (0–0.04)	0.050
Neutrophil-to-lymphocyte ratio (NLR)			
Median (range)	2.08 (1.02–12.75)	2.91 (0.99–11.89)	0.085
Lymphocyte-to-monocyte ratio (LMR)			
Median (range)	3.69 (1.00–9.75)	2.40 (1.05–6.49)	0.024
Neutrophil-to-monocyte ratio (NMR)			
Median (range)	7.24 (3.27–20.35)	8.01 (2.27–26.50)	0.837
Hemoglobin (g/l)			
Median (range)	133.50 (83.00–158.00)	137.00 (96.00–168.00)	0.294
Hematocrit (HCT) (l/l)			
Median (range)	0.40 (0.26–0.46)	0.39 (0.30–0.50)	0.329
Mean corpuscular volume (MCV) (fl)			
Median (range)	92.20 (77.70–98.00)	85.90 (63.30–96.10)	<0.001
Red cell distribution width (RDW) (%)			
Median (range)	12.95 (11.80–20.80)	13.10 (11.80–18.00)	0.712
Platelet (×10^9^)			
Median (range)	212.50 (123.00–656.00)	235.00 (116.00–501.00)	0.554
Platelet-to-lymphocyte ratio (PLR)			
Median (range)	115.71 (51.25–381.25)	136.40 (74.09–357.89)	0.006
Thrombocytocrit (g/l)			
Median (range)	0.21 (0.12–0.42)	0.24 (0.15–0.50)	0.303
CEA (ng/ml)			
Median (range)	2.85 (0.30–9.50)	4.90 (0.60–27.70)	0.002
AFP (ng/ml)			
Median (range)	2.53 (1.36–11.90)	2.70 (1.60–10.53)	0.727
CA199 (ng/ml)			
Median (range)	12.50 (0.80–2153.70)	7.60 (0.80–4269.70)	0.864

**Table 2:** Univariate analyses.

Variable	OR	95% CI	*p*
Sex	0.672	0.160–2.828	0.588
Age	0.969	0.928–1.011	0.147
Differentiation	0.175	0.067–0.458	<0.001
White blood cell count	1.115	0.861–1.443	0.411
Neutrophil granulocyte	1.117	0.849–1.471	0.429
Monocyte	8.641	0.472–158.074	0.146
Lymphocyte	0.350	0.140–0.877	0.025
Eosinophilic granulocyte	0.044	0–5.122	0.198
Basophilic granulocyte	0	0–14.046	0.066
NLR	1.303	0.939–1.808	0.113
LMR	0.672	0.465–0.971	0.034
NMR	1.013	0.902–1.137	0.833
Hemoglobin	1.016	0.987–1.046	0.290
HCT	216.768	0.005–9.522 × 10^6^	0.324
MCV	0.799	0.689–0.926	0.003
RDW	0.938	0.673–1.308	0.707
Platelet	1.002	0.996–1.008	0.550
PLR	1.011	1.002–1.020	0.011
Thrombocytocrit	82.374	0.018–3.703 × 10^5^	0.304
CEA	1.214	1.030–1.430	0.020
AFP	0.954	0.738–1.234	0.722
CA199	1	0.999–1.001	0.861

**Table 3:** Multivariate analyses.

Variable	OR	95% CI	*p*
Differentiation	0.056	0.004–0.889	0.041
Lymphocyte	0.247	0.007–9.011	0.446
LMR	0.385	0.103–1.438	0.156
MCV	0.615	0.401–0.942	0.025
PLR	0.995	0.971–1.019	0.670
CEA	1.639	1.126–2.386	0.010
